# Prediction of the risk of cytopenia in hospitalized HIV/AIDS patients using machine learning methods based on electronic medical records

**DOI:** 10.3389/fpubh.2023.1184831

**Published:** 2023-07-28

**Authors:** Liling Huang, Bo Xie, Kai Zhang, Yuanlong Xu, Lingsong Su, Yu Lv, Yangjie Lu, Jianqiu Qin, Xianwu Pang, Hong Qiu, Lanxiang Li, Xihua Wei, Kui Huang, Zhihao Meng, Yanling Hu, Jiannan Lv

**Affiliations:** ^1^Guangxi Clinical Center for AIDS Prevention and Treatment, Chest Hospital of Guangxi Zhuang Autonomous Region, Liuzhou, Guangxi, China; ^2^School of Information and Management, Guangxi Medical University, Nanning, Guangxi, China; ^3^Nanning Center for Disease Control and Prevention, Nanning, Guangxi, China; ^4^Center for Genomic and Personalized Medicine, Guangxi key Laboratory for Genomic and Personalized Medicine, Guangxi Collaborative Innovation Center for Genomic and Personalized Medicine, Guangxi Medical University, Nanning, Guangxi, China; ^5^Institute of Life Sciences, Guangxi Medical University, Nanning, Guangxi, China; ^6^Basic Medical College of Guangxi Medical University, Nanning, Guangxi, China; ^7^Department of Infection, Affiliated Hospital of the Youjiang Medical University for Nationalities, Baise, Guangxi, China

**Keywords:** HIV, cytopenia, anemia, thrombocytopenia, leukopenia, electronic medical records, machine learning

## Abstract

**Background:**

Cytopenia is a frequent complication among HIV-infected patients who require hospitalization. It can have a negative impact on the treatment outcomes for these patients. However, by leveraging machine learning techniques and electronic medical records, a predictive model can be developed to evaluate the risk of cytopenia during hospitalization in HIV patients. Such a model is crucial for designing a more individualized and evidence-based treatment strategy for HIV patients.

**Method:**

The present study was conducted on HIV patients who were admitted to Guangxi Chest Hospital between June 2016 and October 2021. We extracted a total of 66 clinical features from the electronic medical records and employed them to train five machine learning prediction models (artificial neural network [ANN], adaptive boosting [AdaBoost], k-nearest neighbour [KNN] and support vector machine [SVM], decision tree [DT]). The models were tested using 20% of the data. The performance of the models was evaluated using indicators such as the area under the receiver operating characteristic curve (AUC). The best predictive models were interpreted using the shapley additive explanation (SHAP).

**Result:**

The ANN models have better predictive power. According to the SHAP interpretation of the ANN model, hypoproteinemia and cancer were the most important predictive features of cytopenia in HIV hospitalized patients. Meanwhile, the lower hemoglobin-to-RDW ratio (HGB/RDW), low-density lipoprotein cholesterol (LDL-C) levels, CD4^+^ T cell counts, and creatinine clearance (Ccr) levels increase the risk of cytopenia in HIV hospitalized patients.

**Conclusion:**

The present study constructed a risk prediction model for cytopenia in HIV patients during hospitalization with machine learning and electronic medical record information. The prediction model is important for the rational management of HIV hospitalized patients and the personalized treatment plan setting.

## 1. Background

The human immunodeficiency virus (HIV) not only cause damage to the function of the immune system, but also have a negative impact on the body’s hematopoietic system (1). Cytopenia is one of the common complications of HIV infection (2) and the common types are anemia, thrombocytopenia and leucopenia. Within the HIV patients, anemia is an independently influential factor in both the acceleration of disease progression and the decline in quality of life (3). The prevalence of anemia ranges from 1.3 to 95% (4). Currently there are relatively few reports on the prevalence of leukopenia and thrombocytopenia and their associated factors. The most common type of leukopenia is neutropenia. Neutropenia affects 5 to 30% of patients in the early stages of HIV infection. Whereas in patients with late-stage HIV infection, the prevalence of neutropenia can reach 57 to 76% (5–7). The prevalence of thrombocytopenia among HIV patients ranges from 4.1 to 40% (8). Cytopenia may negatively affect outcomes of treatment and accelerate disease progression in patients with HIV (9). The causes of cytopenia in HIV patients are complicated. Currently reported factors that have been correlated with the occurrence of cytopenias in HIV patients include the direct effects of HIV infection, the effects of drug therapy and OIs (10–12). And CD4^+^ T cell counts as a marker of acquired immunodeficiency syndrome (AIDS) progression have also been proven to correlate with cytopenia (13).

Machine learning has had a wide range of applications in medicine in recent years, such as cancer diagnosis (14), medical imaging (15) and death prediction (16). Numerous machine learning algorithms have demonstrated their potential for application to large-scale biomedical and patient datasets. Machine learning can balance the deviation and variance of data. Machine learning can be utilized on datasets containing numerous multidimensional variables to identify high-dimensional, non-linear relationships between clinical features for the purpose of data-driven outcome prediction. This approach overcomes certain limitations of current risk prediction analysis methods. Machine learning models for medical big data based on electronic medical records will support doctors in clinical diagnosis and management.

Cytopenia continues to be a significant concern in numerous countries with limited resources. The severity of cytopenia and its associated factors can impact the effectiveness of highly active antiretroviral therapy (HAART). However, this issue has not received enough attention in many developing countries. Most reports on the prevalence and associated factors of cytopenias come from regions with high AIDS prevalence and developed countries. These data may be quite different from other regions in terms of patient characteristics, cytopenic status, and HAART, etc. (17–20). The main aim of the present study was to construct a predictive model that accurately predicts whether cytopenia would occur during hospitalization in people with HIV. To develop more appropriate treatment plans for HIV patients, it is essential to understand the profile of cytopenias and the relevant factors (21). However, there have been few reports on cytopenias among HIV patients in China. Thus, gaining insight into the risk factors that contribute to cytopenia in patients with HIV and developing an accurate predictive model for cytopenia could facilitate early intervention and prevent its progression in this patient population. For clinicians, the model could be used to screen HIV patients who may experience cytopenia in the future, and thus take a more appropriate treatment approach.

## 2. Materials and methods

### 2.1. Data collection

This study was carried out at Guangxi Chest Hospital. Guangxi Chest Hospital is located in Liuzhou, Guangxi. The hospital is the regional designated hospital for the treatment of serious infectious diseases. The study was carried out between June 2016 and October 2021 and enrolled a total of 6,220 hospitalized HIV infected patients. Through the hospital electronic medical record system identifying HIV patients with cytopenia. People with HIV who did not suffer from cytopenia on their admission were included as study participants. The diagnostic criteria for anemia is the same as that of the World Health Organization (WHO). A hemoglobin level < 110 g/L (women) or < 120 g/L (men) is defined as anemia. Anemia is graded as severe (hemoglobin <60 g/L), moderate (hemoglobin 60–89 g/L) and mild (hemoglobin 90–119 g/L for men or 90–109 g/L for women). Compared to anemia, leukopenia and thrombocytopenia do not have universally accepted cut-off values. We defined them using criteria that have been used in other studies (7, 22). The criterion for leukopenia was total leukocytes <4.0 × 103/uL. Platelet counts <150 × 103/uL were considered to be thrombocytopenia. The classification criteria for mild thrombocytopenia, moderate thrombocytopenia and severe thrombocytopenia were 100–150 × 103/uL, 50–100 × 103/uL and less than 50 × 103/uL, respectively, Gunda et al. (9). If a patient has multiple admissions to hospital, the data of the most recent admission will be included as a priority. The results of laboratory tests on the patient’s blood first collected on admission to hospital were included in the study. The patient was discharged from hospital or died during hospitalization then observation was stopped. Patients younger than 18 years old, patients who received radiation therapy within the past 45 days, and pregnant women were excluded from the study. Because the underlying conditions of these patients themselves may induce or exacerbate cytopenias. All of the patients were confirmed to be HIV-positive by enzyme-linked immunosorbent assay and immunoblot detection laboratory tests, and the diagnosis was consistent with national HIV diagnostic criteria.

### 2.2. Data preprocessing

We extracted sociodemographic and clinical information, as well as blood examination records, from the medical electronic record system of Guangxi Chest Hospital to construct a structured dataset for the study participants. The structured dataset included 66 variables: 13 clinical comorbidity/co-infection variables (tuberculosis, pneumocystis, candida infection, cryptococcus, herpesvirus, cytomegalovirus, pneumonia, electrolyte disorders, hepatitis (B or C), hypoproteinemia, diabetes, hypertension, and cancer) 6 demographic indicators (gender, age, ethnicity, marital status, actual days in hospital and residence) 47 laboratory indicators (CD8^+^ T cell count, CD4^+^ T cell count and levels of ALP, ALT, AST, CEA, etc.).Excluding variables with missing data greater than 15%. Used Random Forest to fill in the missing values for the structured dataset (23). All the above data processing steps were done by the numpy, pandas and sklearn packages of Python 3.8.6.

### 2.3. Model construction and evaluation

Whether cytopenia had occurred in HIV patients at hospital discharge was used as a outcome of the prediction model. The data was divided randomly into two datasets using Scikit-learn, a Python package (24). 80% of the data was utilized for training the machine learning models and adjusting their parameters. 20% of the data were used to test the models and fine-tune the hyperparameters. We used five machine learning classifiers (artificial neural network (ANN), adaptive boosting (AdaBoost), k-nearest neighbour (KNN), support vector machine (SVM) and decision tree (DT)) to create five models for predicting outcomes. The five machine learning classification prediction models were all constructed based on the sklearn package from Python 3.8.6.

The predictive ability of the prediction models was evaluated using the area under the receiver operating characteristic (ROC) curve (AUC), specificity, accuracy, sensitivity, and F1 scores. The evaluation indicators were varied from 0 to 1, corresponding to the worst and best scores, respectively. Using these metrics together allowed a more comprehensive evaluation and comparison of the classification effectiveness of different machine learning methods. The prediction model with the most effective performance evaluation indicators was selected as the final model. To explain the outcome of the best-performing predictive model, we utilized the Shapley additive interpretation (SHAP) to calculate the contribution of each feature to the predicted outcome (25).

### 2.4. Statistical analysis

The analysis of data was conducted with Python version 3.8.6 and SPSS version 24 statistical software package (SPSS Inc., Chicago, IL). The descriptive statistics such as percentage, mean, median, IQR, and standard deviation were used as appropriate. Student’s *t*-test was used to compare normally distributed continuous variables, while the Mann–Whitney U test was used for non-normally distributed continuous variables. The chi-square test was utilized to compare categorical variables. The tests were two-sided, and statistical significance was defined as *p* values less than 0.05.

### 2.5. Ethical statement

The Human Research Ethics Committee of Guangxi Medical University (ethical approval number: 20210172) and the Medical Ethics Committee of Chest Hospital (ethical approval number: 2022–011) approved this study. Informed consent was waived after review by the Chest Hospital Medical Ethics Committee. Patient information was de-identified, and confidentiality was maintained throughout the study.

## 3. Result

### 3.1. Sociodemographic characteristics of study participants

**Figure 1:**
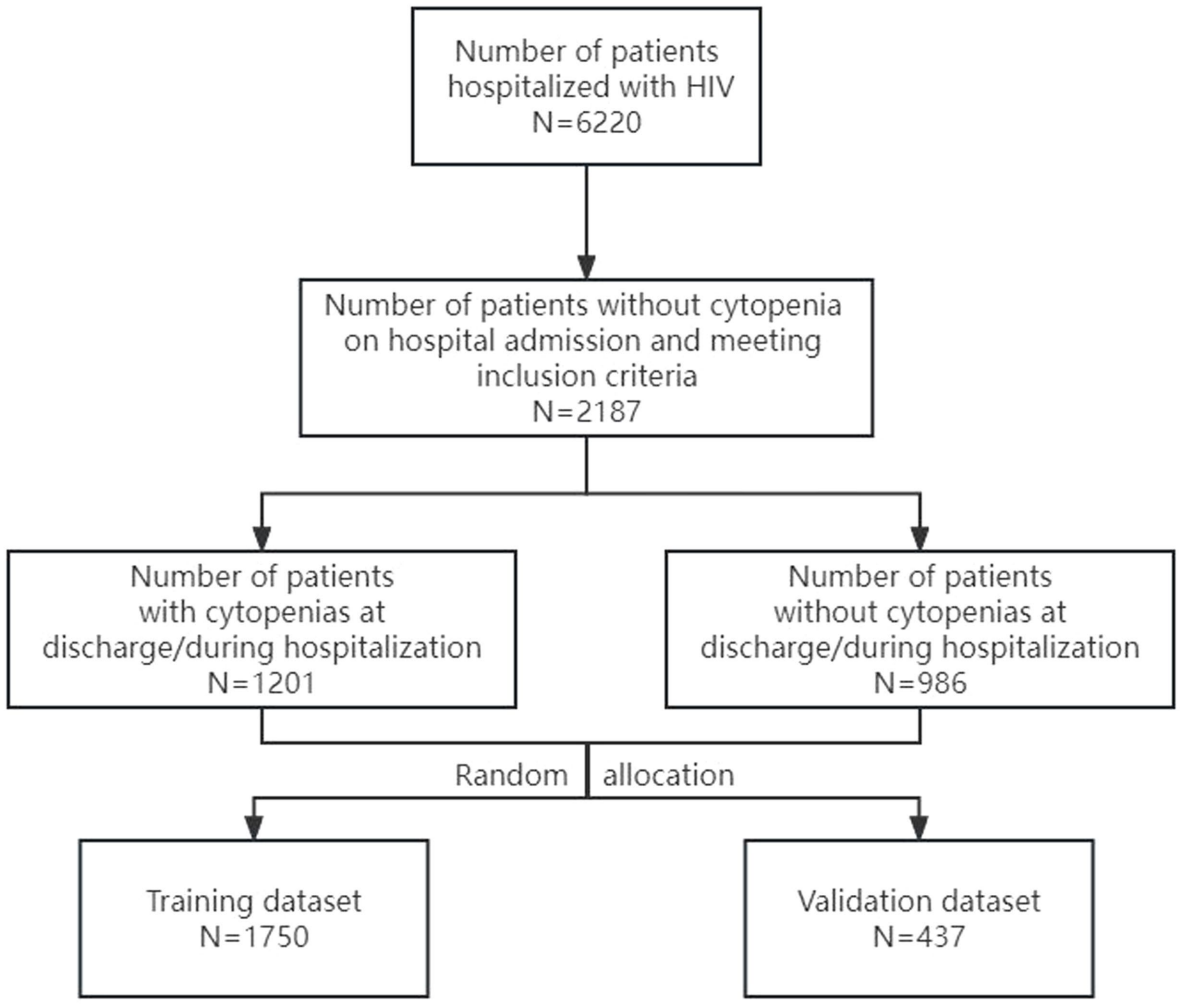
Flow diagram of the selection of participants included in the present study.

In this research, the prevalence of cytopenia in hospitalized HIV patients was 19.3% (1,201/6220). The study included 2,187 qualified people living with HIV. Figure 1 showed the selection process for the patients included in the present study. The study participants had a median age of 56 years (interquartile range (IQR): 45–66 years). The median number of days in hospital for study participants was 21 days (interquartile range (IQR): 12–33 days). Among the 2,187 study participants, 1,686 (77.1%) were male, 1,673 (76.5%) were from rural areas and 1,296 (59.3%) were married. Over half (55.0%) of the sample were from ethnic minority groups, with the Zhuang ethnic group comprising the majority (48.0%). The cytopenia and non-cytopenia groups differed significantly in demographic characteristics, including gender, ethnicity, marital status, and residence address (*p* < 0.05). The essential features of the study participants were listed in Table 1.

**Table 1:** Sociodemographic characteristics of HIV patients included in study.

Variables	Total (*n* = 2,187)	Outcome		Statistic	*p*-value
		Non-cytopenias (*n* = 986)	Cytopenias (*n* = 1,201)		
Age, years	56 (45,66)	56 (45,66)	55 (44,66)	−1.023	0.306
Days in hospital, days	21 (12,33)	22 (13, 32)	21 (12, 33)	−0.102	0.919
Sex				8.718	**0.003**
Female	501 (22.9%)	197 (39.3%)	304 (60.7%)		
Male	1,686 (77.1%)	789 (46.8%)	897 (53.2%)		
Ethnicity				8.586	**0.014**
Han	986 (45.0%)	415 (42.1%)	571 (57.9%)		
Zhuang	1,049 (48.0%)	507 (48.3%)	542 (51.7%)		
Other (minority)	152 (7.0%)	64 (42.1%)	88 (57.9%)		
Marital Status				7.289	**0.026**
Unmarried	485 (22.2%)	204 (42.1%)	281 (57.9%)		
Married	1,296 (59.3%)	615 (47.5%)	681 (52.5%)		
Divorce and widowhood	406 (18.6%)	167 (41.1%)	239 (58.9%)		
Residence				6.803	**0.009**
Urban	514 (23.5%)	206 (40.1%)	308 (59.9%)		
Rural	1,673 (76.5%)	780 (46.6%)	893 (53.4%)		

Numbers in bold represent significant groups (*p* < 0.05).

### 3.2. Clinical and laboratory characteristics of study participants

The most common clinical complication/co-infection in hospitalized HIV patients was Candida infection (56.8%, 1243/2187), followed by hypoproteinaemia (48.0%, 1049/2187), pneumonia (47.1%, 1030/2187), and tuberculosis (47.1%, 1030/2187). The prevalence of cytopenia was as high as 76.0% (190/250) in HIV patients with electrolyte disturbances, which was higher than 52.2% (1,011/1937) in HIV patients without electrolyte disturbances. The prevalence of cytopenia was 72.4% (760/1049) in HIV patients with hypoproteinaemia, which was higher than 38.8% (441/1138) in HIV patients without hypoproteinaemia. Table 2 showed detailed information about clinical complications/co-infections of the study participants.

**Table 2:** Characteristics of clinical complications/co-infections in HIV-positive patients included in study.

Variables	Total (*n* = 2,187)	Outcome		Statistic	*p*-value
		Non-cytopenias (*n* = 986)	Cytopenias (*n* = 1,201)		
Pneumonia				0.797	0.372
No	1,157 (52.9%)	532 (46.0%)	625 (54.0%)		
Yes	1,030 (47.1%)	454 (44.1%)	576 (55.9%)		
Tuberculosis				0.519	0.471
No	1,157 (52.9%)	530 (45.8%)	627 (54.2%)		
Yes	1,030 (47.1%)	456 (44.3%)	574 (55.7%)		
Pneumocystis				1.266	0.260
No	1,653 (75.6%)	734 (44.4%)	919 (55.6%)		
Yes	534 (24.4%)	252 (47.2%)	282 (52.8%)		
Candidiasis				9.435	0.002
No	944 (43.2%)	461 (48.8%)	483 (51.2%)		
Yes	1,243 (56.8%)	525 (42.2%)	718 (57.8%)		
Cryptococcus				12.046	0.001
No	2093 (95.7%)	960 (45.9%)	1,133 (54.1%)		
Yes	94 (4.3%)	26 (27.7%)	68 (72.3%)		
Herpesvirus				2.055	0.152
No	1972 (90.2%)	899 (45.6%)	1,073 (54.4%)		
Yes	215 (9.8%)	87 (40.5%)	128 (59.5%)		
Cytomegalovirus				10.781	0.001
No	2020 (92.4%)	931 (46.1%)	1,089 (53.9%)		
Yes	167 (7.6%)	55 (32.9%)	112 (67.1%)		
Electrolyte disturbances				50.684	<0.001
No	1937 (88.6%)	926 (47.8%)	1,011 (52.2%)		
Yes	250 (11.4%)	60 (24.0%)	190 (76.0%)		
Hypoproteinemia				250.351	<0.001
No	1,138 (52.0%)	697 (61.2%)	441 (38.8%)		
Yes	1,049 (48.0%)	289 (27.6%)	760 (72.4%)		
Hepatitis B/Hepatitis C				6.564	0.01
No	1903 (87.2%)	878 (46.1%)	1,025 (53.9%)		
Yes	284 (12.8%)	108 (38.0%)	176 (62.0%)		
Hypertension				0.272	0.602
No	2036 (93.1%)	921 (45.2%)	1,115 (54.8%)		
Yes	151 (6.9%)	65 (43.0%)	86 (57.0%)		
Diabetic Mellitus				0.792	0.373
No	2073 (94.8%)	930 (44.9%)	1,143 (55.1%)		
Yes	114 (5.2%)	56 (49.1%)	58 (50.9%)		
Cancer				214.099	<0.001
No	1954 (89.3%)	986 (50.5%)	968 (49.5%)		
Yes	233 (10.7%)	0 (0.0%)	233 (100.0%)		

We evaluated the median levels of important indicators in both cytopenic and non-cytopenic groups of patients with HIV. The hemocytopenic group had lower levels of CD4^+^ T cell count, CD45^+^ T cell count, CD3^+^ T cell count, cholinesterase (CHE), creatinine clearance (Ccr), prealbumin (PA) and total cholesterol (CHOL). There were also some laboratory indicators of interest that were significantly different, such as serum cystatin (Cys-C), triglycerides (TG) and chlorine (Cl). Detailed characteristics of the laboratory indicators were shown in Table 3.

**Table 3:** Characteristics of the laboratory measures of the HIV patients included in the study.

Variables	Total (*n* = 2,187)	Outcome		*p*-value
		Non-cytopenias (*n* = 1,108)	Cytopenias (*n* = 1,079)	
CD3^+^ T cell count (cell/ul)	493 (274,814)	550 (315,899)	456 (242,745)	<0.001
CD4/CD8 ratio	0.16 (0.06,0.36)	0.17 (0.07,0.39)	0.15 (0.06,0.33)	0.004
CD4^+^ T cell count (cell/ul)	63 (18,167)	76 (20,214)	54 (15,143)	<0.001
CD45^+^ T cell count (cell/ul)	743 (433,1,194)	843 (491,1,321)	681 (378,1,061)	<0.001
CD8^+^ T cell count (cell/ul)	374 (217,617)	402 (245,665)	352 (191,578)	<0.001
(1–3)-β-D Glucan (pg/ml)	9.3 (7.3,77.4)	9.2 (7.2,58.0)	9.5 (7.4,105.4)	0.02
CEA (ng/mL)	3.9 (1.7,26.3)	4.1 (1.8,28.3)	3.8 (1.7,25.1)	0.162
ALB (g/L)	31.0 (26.5,35.4)	32.6 (27.8,36.6)	30 (25.3,34.1)	<0.001
HCO3std (mmol/L)	25.1 (22.5,27.1)	25.5 (23.3,27.2)	24.8 (21.6,26.9)	<0.001
CHE (U/L)	4643.0 (3165.0,6358.0)	5194.1 (3718.8,6936.3)	4233.0 (2804.0,5786.5)	<0.001
LDL-C (mmol/L)	2.2 (1.6,2.7)	2.4 (1.8,2.9)	2.0 (1.5,2.5)	<0.001
AMY (U/L)	93.0 (68.0,125.0)	91.9 (68.0,120.2)	94.0 (67.0,129.4)	0.311
Ca (mmol/L)	2.1 (1.9,2.2)	2.1 (2.0,2.2)	2.1 (1.8,2.2)	<0.001
TG (mmol/L)	1.4 (1.1,2)	1.5 (1.1,2.1)	1.4 (1.0,2.0)	0.041
HDL-C (mmol/L)	0.8 (0.6,1.1)	0.9 (0.7,1.1)	0.8 (0.5,1.1)	<0.001
GGT (U/L)	73 (39,139)	69.0 (38.8,127.0)	79.0 (40.0,150.0)	0.032
ALT (U/L)	20 (12,35)	21.5 (13.0,37.0)	19.0 (11.0,34.0)	0.001
AST/ALT	1.5 (1.0,2.2)	1.4 (0.9,2.0)	1.6 (1.1,2.3)	<0.001
AST (U/L)	28 (20,44)	27 (20,41)	29 (19,46)	0.306
Cys-C (mg/L)	1.19 (1.00,1.53)	1.14 (0.98,1.41)	1.24 (1.03,1.65)	<0.001
CREA (umol/L)	69 (56,88)	69 (56,85)	70 (55,92)	0.173
Ccr (ml/min)	63.2 (48.2,76)	66.2 (52.6,77.7)	60.5 (44.4,74.1)	<0.001
CK (U/L)	55.0 (34.0,95.5)	57.0 (36.0,99.0)	53.0 (33.0,92.0)	0.04
K (mmol/L)	3.7 (3.3,4.1)	3.7 (3.3,4.0)	3.7 (3.3,4.1)	0.928
IBIL (umol/L)	3.6 (2.4,5.6)	3.6 (2.4,5.6)	3.5 (2.4,5.6)	0.967
ALP (U/L)	99 (70,143)	92.0 (68.0,134.0)	105.0 (73.0,151.5)	<0.001
Cl (mmol/L)	102.9 (100.0,105.8)	102.0 (99.0,105.0)	102.9 (100.0,106.8)	<0.001
Mg (mmol/L)	0.8 (0.7,0.8)	0.77 (0.70,0.84)	0.75 (0.67,0.82)	<0.001
Na (mmol/L)	136 (133,139)	136 (133,139)	136 (133,139)	0.545
UREA (mmol/L)	4.1 (3.0,5.9)	4.0 (2.9,5.4)	4.3 (3.0,6.3)	0.001
UA (umol/L)	299 (217,416)	303 (227,430)	295 (211,409)	0.011
Glu (mmol/L)	6.8 (5.5,8.5)	6.9 (5.6,8.8)	6.7 (5.5,8.4)	0.008
PA (mg/L)	151 (91,218)	170 (109,237)	137 (78,205)	<0.001
GLOB (g/L)	37.4 (31.4,43.6)	36.9 (31.4,42.6)	37.8 (31.6,44.4)	0.031
LDH (U/L)	227 (179,307)	224 (179,296)	230 (179,315)	0.238
ADA (U/L)	22.6 (15.1,31.5)	22.3 (15.5,30.0)	22.8 (14.4,32.9)	0.359
DBIL (umol/L)	2.8 (1.7,5.6)	2.6 (1.6,4.8)	3.0 (1.7,6.5)	0.001
CHOL (mmol/L)	3.6 (2.9,4.4)	3.9 (3.1,4.6)	3.4 (2.7,4.1)	<0.001
TBIL (umol/L)	6.6 (4.4,11.1)	6.5 (4.4,10.3)	6.7 (4.5,12.0)	0.031
TBA (umol/L)	7.6 (3.8,15)	6.8 (3.5,13.2)	8.3 (4.1,17.0)	<0.001
TP (g/L)	69.2 (61.6,75.6)	70.1 (62.9,76.0)	68.5 (60.6,75.4)	0.001

### 3.3. Feature selection, model construction and evaluation

Using the sklearn package and the pandas package in Python 3.8.6 to achieve feature filtering of the data. We used recursive feature elimination (RFE) with random forest to select input features for a predictive model aimed at predicting the occurrence of cytopenia in HIV patients during hospitalization. Finally, 12 variables were selected from 66 variables as predictors of the risk of cytopenia in HIV patients. Among the 12 included indicators, 9 were laboratory examination indicators, including CD4^+^ T cell count, serum cystatin (Cys-C), standard bicarbonate (HCO3std), low-density lipoprotein cholesterol (LDL-C), creatinine clearance (Ccr), chloride (Cl), glutamyltransferase (GGT), monocytes-to-lymphocites ratio (Mono/Lymph) and hemoglobin-to-RDW ratio (HGB/RDW), 3 clinical comorbidity/co-infection including electrolyte disturbances, hypoproteinemia and cancer.

**Figure 2:**
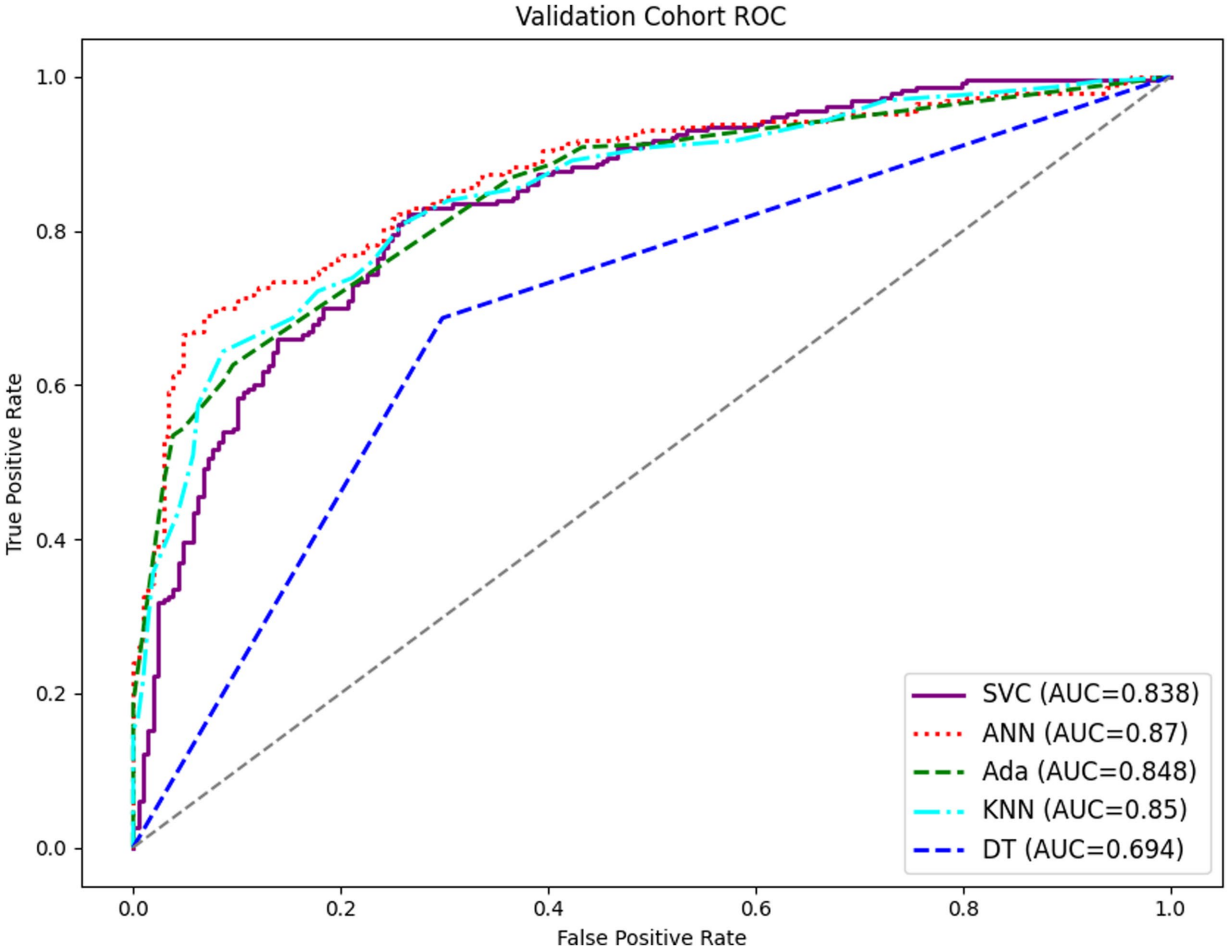
Evaluation of five machine learning algorithms based on the AUC of ROC curves.

The prediction models for the development of cytopenia in HIV patients during the hospitalization were constructed based on 12 features from the feature selection results. Table 4 displayed the prediction performance of the prediction models generated by the five machine learning algorithms. The ANN model demonstrated the highest sensitivity and specificity and therefore exhibited superior predictive power compared to other models. Figure 2 showed the ROC curves for the five models, with the ANN model displaying the most favorable results.

**Table 4:** Performance of predictive models built by five machine learning algorithms.

Models	AUC	Sensitivity	Specificity	Accuracy	F1	Precision
SVC	0.844	0.730	0.787	0.758	0.753	0.779
KNN	0.831	0.735	0.767	0.751	0.740	0.745
ANN	0.858	0.745	0.798	0.772	0.766	0.788
AdaBoost	0.852	0.745	0.788	0.767	0.759	0.774
DT	0.647	0.634	0.661	0.648	0.624	0.615

### 3.4. Explanation of risk factor

**Figure 3:**
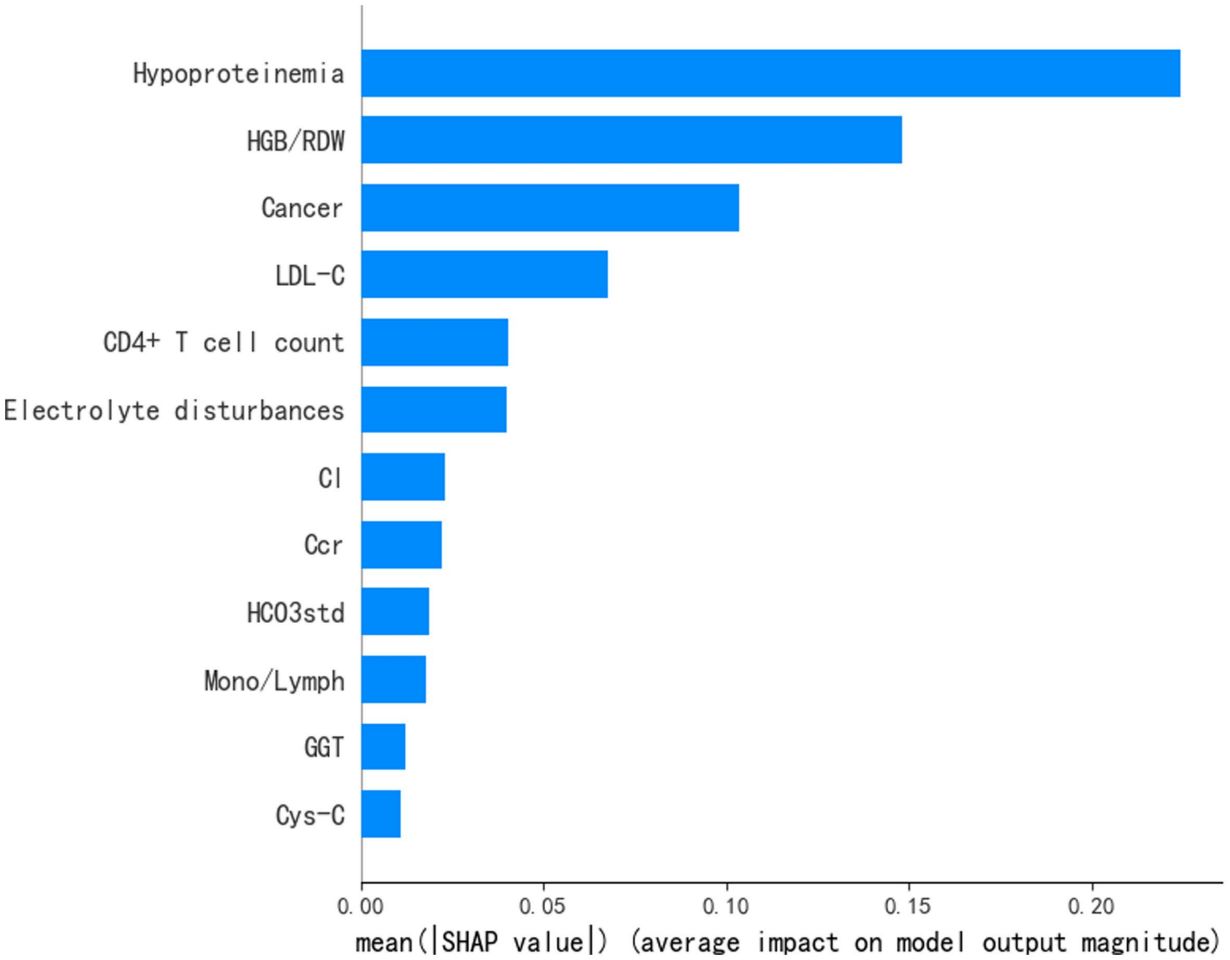
Importance matrix plot of the ANN model showing the contribution of each clinical feature to the predictive model of cytopenia in hospitalized HIV patients.

**Figure 4:**
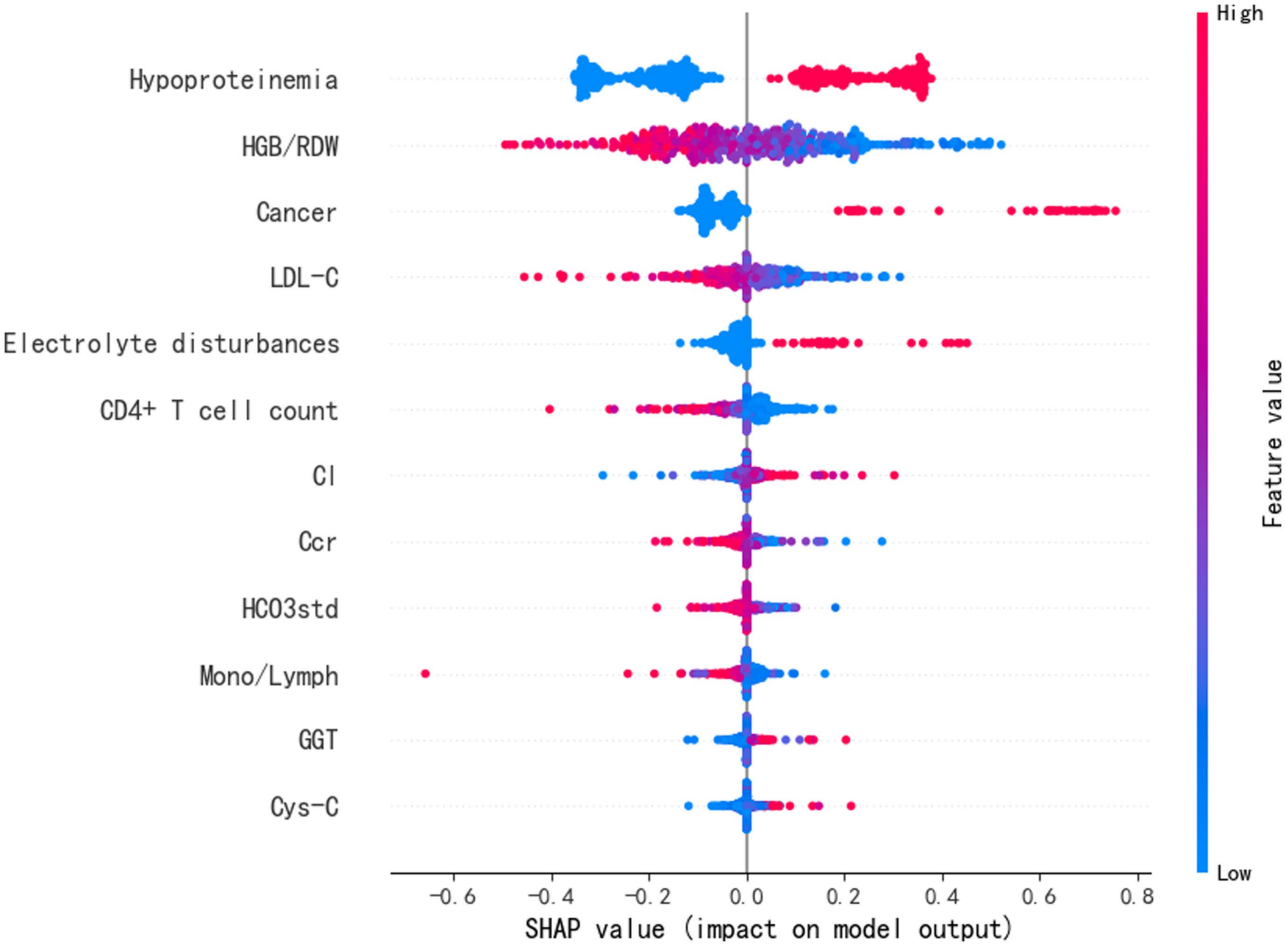
SHAP summary plot of the top 12 clinical features of the ANN model.

To better comprehend how the features integrated into the ANN prediction model contribute to the prediction results, we computed the SHAP values for each individual feature. The ANN prediction model generates a predictive value for each predicted sample. The SHAP value is a numerical score assigned to each feature in a given sample, indicating the degree of impact each feature has on the outcome and whether it is a positive or negative influence. The importance matrix diagram for the ANN model was shown in Figure 3. The importance matrix ranks the features that affect cytopenias in hospitalized HIV patients, from most to least important. The importance matrix ranking results for the ANN prediction model were hypoproteinemia, HGB/RDW, cancer, LDL-C, CD4^+^ T cell count, electrolyte disturbance, Cl, Ccr, HCO3std, Mono/Lymph, GGT and Cys-C. The SHAP summary plot showed how each variable had an impact on the predicted outcome of the occurrence of cytopenia in hospitalized HIV patients (Figure 4). Each patient was assigned a point, and features were color-coded based on attribute values, with red indicating higher values and blue indicating lower values. According to the SHAP summary plots, hypoproteinemia and cancer were identified as the most significant features. In hospitalized patients with HIV, these two features were strongly and positively correlated with cytopenia. HIV patients presenting with these two clinical comorbidities were at significantly increased risk of developing cytopenia during hospitalization compared to HIV patients not presenting with these two comorbidities. HGB/RDW, LDL-C, CD4^+^ T cell count, Ccr, HCO3std and Mono/Lymph also had a significant effect on the occurrence of cytopenia in hospitalized patients with HIV. The risk of cytopenia increases as the value of these features decreases. The higher the value of Cl, GGT and Cys-C, the greater the risk of cytopenia. Hospitalized HIV patients with electrolyte disorders were more likely to develop cytopenia.

## 4. Discussion

This study conducted a retrospective analysis on a large sample size and identified Candida infection, hypoproteinemia, tuberculosis and pneumonia as the most frequent complications among hospitalized patients with HIV. This finding was similar to previous reports (26). The current study employed machine learning techniques and clinical features readily obtainable from electronic medical records to develop a predictive model for cytopenia risk in HIV patients during hospitalization. We evaluated and compared the predictive capabilities of five distinct machine learning models. The results showed that ANN models have the highest sensitivity and specificity. ANN model is widely used in clinical detection and pathology identification due to the good performance it has shown in recognition (27, 28). In comparison to other machine learning models, ANN are able to effectively process non-linear relationships, which is important in many real-world problems (29). ANN consist of multiple neurons and layers that enable them to learn and represent very complex relationships and have better capabilities for implicit pattern and feature extraction in data. The hidden layer structure of ANN enables them to capture and represent complex relationships between input features, thus better adapting to different types of data (30). As far as the authors know, the current research is the first published study to use ANN models to predict the occurrence of cytopenia in HIV patients during hospitalization.

The combination of electronic medical records and machine learning has contributed to the development of complex prediction models (31, 32). To enhance the transparency of the model’s prediction process, we employed the SHAP method to compute the contribution of individual variables to the model’s predicted outcome. The results showed that hypoproteinemia and cancer were important factors influencing the occurrence of cytopenia during hospitalization of HIV patients. We also identified HGB/RDW, LDL-C, CD4^+^ T cell count and Ccr were the variables that had a greater impact.

Previous studies have demonstrated that hypoproteinemia is a potential predictor of disease progression and mortality among individuals with HIV (33). A cohort study in West Africa that investigated the nutritional status of HIV patients who received HAART for 1 year reported that low albumin was associated with anemia (34). It is not coincidental that serum albumin levels have been claimed to be independently associated with severe anemia and could influence mortality and the outcome of HAART in HIV patients (35). There is growing evidence that hypoproteinemia has a dramatic impact on cytopenia in HIV patients, particularly anemia. There are two possible reasons why people with cancer are more likely to develop anemia; cancer causes difficulty in the production of red blood cells and shortens the survival time of red blood cells (36). Furthermore, anti-cancer treatments may harm healthy blood cells. Our study discovered that hypoproteinemia and cancer were significant factors contributing to cytopenia in HIV patients during hospitalization.

After analyzing all features included in the model, we found that HGB/RDW, LDL-C, CD4^+^ T cell count, and Ccr had the great impact on predicting the risk of cytopenia during hospitalization in HIV patients. Specifically, lower levels of HGB/RDW, LDL-C, CD4^+^ T cell count, and Ccr were associated with an increased risk of cytopenia. HGB/RDW as a new comprehensive biomarker has gradually attracted widespread attention. The lower HGB/RDW levels have been demonstrated to be associated with cancer development and poor prognosis (37, 38). In the present case, HIV patients with lower levels of HGB/RDW had a higher risk of cytopenia during hospitalization. The lower HGB/RDW may represent abnormal erythrocyte homeostasis and deformed erythrocytes, leading to disturbed blood flow in the microcirculation (39), which may have contributed to the increased susceptibility of people living with HIV to cytopenias during hospitalization. Hemoglobin and RDW are easily accessible laboratory examination indicators. But HGB/RDW is rarely focused on during HIV treatment. The results of the present study showed that HGB/RDW is strongly associated with the development of cytopenias in people living with HIV and deserves greater attention.

Low LDL-C is often associated with long-term vegetarian diet (40), liver disease (41) and drug therapy (42). Low LDL-C has also been reported to be associated with chronic anemia (43). However, LDL-C has not been of particular concern in previous studies about risk factors associated with cytopenia in HIV patients. Although the mechanism of how lower LDL-C leads to cytopenia is not clear, there are some possible explanations. Possible explanations include erythrocyte fragility due to low cholesterol levels in the erythrocyte membrane (44), as well as LDL-related platelet activation and tissue factor expression (45). But the mechanism of how low LDL-C leads to cytopenia needs more further research to prove it.

As with previous studies, our research found that low CD4^+^ T cell counts are a risk factor for cytopenia in HIV patients. CD4^+^ T cell counts are closely correlated with HIV disease progression, and lower counts are typically indicative of advanced disease progression (46). The primary explanation for cytopenia, which results from low CD4^+^ T cell counts in HIV patients, is likely HIV-mediated hematopoietic suppression and direct T cell infection (10). Moreover, research showed that improved CD4^+^ T cell counts after HAART treatment have led to a reduction in the prevalence of cytopenia in HIV patients (47–49), indicating that HIV-related cytopenia is caused by HIV infection and immunosuppression (50).

CCr is a sensitive marker of glomerular damage and an early indicator of kidney impairment. Lower CCr could lead to chronic kidney disease, and the common complications of chronic kidney disease include anemia (51). Concurrently, it has also been claimed that high serum creatinine is a significant predictor of anemia in HIV patients (52). GGT is an important indicator of liver function and an increased GGT level means impaired liver function. And abnormal liver function could cause a cytopenia (53). Both CCr and GGT reflect the organ function of HIV patients and the potential risk of cytopenia in HIV patients, but have not been focused on in previous studies. The levels of Cl, HCO3std and electrolytes provide valuable information about the body’s metabolism, and their disturbances may indicate metabolic issues in HIV patients who were at a high risk of developing cytopenia. Mono/Lymph is demonstrated to be a predictor of the risk of developing tuberculosis in people living with HIV (54). And tuberculosis is one of the factors associated with the development of cytopenia in people living with HIV (9). Although Cys-C levels may be considered clinically insignificant and often overlooked, it is still important for predictive modeling purposes.

The present study used real-world data from electronic medical records to construct an ANN prediction model for predicting the risk of cytopenia in HIV patients during hospitalization using multiple clinical complications and clinical variables. We identified some risk factors associated with cytopenia in HIV patients that have not been focused on in previous studies. Finally, the predictive model can serve as a clinical screening tool to assess the risk of cytopenia in HIV patients during hospitalization, thus facilitating the development of more personalized and rational treatment plans. However, there were certain limitations in our study. Firstly, our study sample was predominantly limited to southern China and thus not indicative of the overall situation of individuals living with HIV throughout China. Secondly, the potential influence of medications and treatment regimens on the study outcomes was not taken into account during the data collection process. Thirdly, the prediction model in this study was not validated for stability using external data. Our model has been internally validated and demonstrates consistent and robust predictive ability for the results explored.

## 5. Conclusion

To sum up, this study utilized electronic medical records to gather demographic information, clinical complications, and laboratory test indicators of HIV patients. These clinical characteristics were then used to construct a predictive model to assess the risk of cytopenia in HIV patients. The predictive model has significant implications for improving the management of HIV patients and tailoring personalized treatment plans.

## Data availability statement

The raw data supporting the conclusions of this article will be made available by the authors, without undue reservation.

## Ethics statement

The Human Research Ethics Committee of Guangxi Medical University (ethical approval number: 20210172) and the Medical Ethics Committee of Chest Hospital (ethical approval number: 2022-011) approved this study. Informed consent was waived after review by the Chest Hospital Medical Ethics Committee. Patient information was de-identified, and confidentiality was maintained throughout the study. Written informed consent for participation was not required for this study in accordance with the national legislation and the institutional requirements.

## Author contributions

YH, JL, LH, JQ, and KZ designed the study and provided the correlative knowledge. YX, LS, YL, YJL, ZM, and KH collected and provided the data. LL, XW, and BX extracted data and cleaned data. BX and LL constructed the prediction model. KZ, LS, YL, and BX generated the figures and tables. YX, YH, HQ, XP, and BX wrote and edited the manuscript. All authors contributed to the article and approved the submitted version.

## Funding

This research was supported by several organizations, including the Guangxi Key Research and Development Program (2021AB12032), Research Projects for High-level Talents in Affiliated Hospital of Youjiang Medical College of Ethnic Minorities in 2022 (R202210308), Guangxi Medical and Health Appropriate Technology Development and Promotion Application Project (S2022042), Nanning Scientific Research and Technology Development Program (20206124), Guangxi Chinese Medicine Appropriate Technology Development and Promotion Project (GZSY22-71), and Major National Science and Technology Projects (2017ZX10202101-001-006). It is important to note that the funding bodies were not involved in the design of the study, data collection, analysis, interpretation or manuscript writing.

## Conflict of interest

The authors declare that the research was conducted in the absence of any commercial or financial relationships that could be construed as a potential conflict of interest.

## Publisher’s note

All claims expressed in this article are solely those of the authors and do not necessarily represent those of their affiliated organizations, or those of the publisher, the editors and the reviewers. Any product that may be evaluated in this article, or claim that may be made by its manufacturer, is not guaranteed or endorsed by the publisher.
